# Orthopaedic trauma care during the COVID-19 Pandemic: the Latin American perspective

**DOI:** 10.1097/OI9.0000000000000114

**Published:** 2021-03-15

**Authors:** Luis G. Padilla-Rojas, Roberto E. López-Cervantes, Leonardo López-Almejo, Israel Gutiérrez-Mendoza, Rafael E. Amadei-Enghelmayer, César A. Pesciallo, José L. Osma-Rueda, Miguel A. Triana-Quijano, Jorge Dos Santos-Silva

**Affiliations:** a Puerta de Hierro Andares Hospital, Zapopan; b CFO (Osteoporosis Fracture Clinic), San Francisco de Asis Hospital Guadalajara; c Star Hospital, Aguascalientes, Ags, Mexico; d Higa San Martín de la Plata and Cuenca Alta SAMIC Hospital, Cañuelas; e Británico Hospital, Buenos Aires, Argentina; f Univerisad Indistrial de Santander, Bucaramanga; g Children's of San José and La Colina Hospital, Bogotá, Colombia; h Institute of Othopaedics and Traumatology Hospital das Clinicas Faculty of Medicine, University of San Paulo, San Paulo, Brazil

**Keywords:** COVID-19, orthopaedic surgery, pandemic, surgical management, trauma care

## Abstract

At first glance, the COVID-19 pandemic and the field of orthopaedics and traumatology do not appear to be related. Although orthopaedists are not considered front-line personnel in the fight against the pandemic, the role of the surgeon as part of the overall health care team is crucial. The specialty of orthopaedics and orthopaedic trauma, due to its extraordinary scope, affects individuals of all ages and timely care affects patients’ long-term function and quality of life. Therefore, positioning the type and timing of care for musculoskeletal injuries and conditions, while maintaining the safety of the patient and healthcare providers, is essential. This article reviews the initial approaches to orthopaedic trauma care during the COVID-19 pandemic as established by 4 representative countries in Latin America: Mexico, Argentina, Colombia, and Brazil.

## 1 Introduction

In December 2019, a new Pneumonia was recognized in Wuhan, China. COVID-19 (SARS[severe acute respiratory syndrome]-CoV-2)^[1]^ was declared an international emergency on January 30,^[1]^ 2020 and then a pandemic on March 11, 2020.^[2]^ We learned that the most common ways of transmission are touching contaminated surfaces, respiratory droplets, and direct contact with a COVID-19 patient or their secretions.^[3]^ Another factor that promotes increasing number of cases is urban mobility, which is related to the daily movements by people in cities, either motorized or not, to accomplish the activities related to work, entertainment, or necessary personal activities.^[4]^

Globally, governments have dictated containment measures to minimize the viral spread. Hospitals are one of the places with greatest risk of transmission. The role of the Orthopaedic surgeon in this pandemic is to minimize the risk of infection and dissemination in patients and health personnel.^[5]^

The main recommendations for managing the pandemic come from places where there have been significant numbers of COVID-19 infections, including England,^[5]^ Indonesia,^[6]^ Portugal,^[7]^ China,^[8]^ and the United States.^[9,10]^ In these countries, the health personnel recognized the importance of using personal protection equipment (PPE), limiting nonurgent and outpatient care, and postponing elective surgeries.^[11]^ This paper will describe the national position on trauma patient care in 4 countries in Latin America during the COVID-19 pandemic.

## 2 Mexico

COVID-19 was first reported in Mexico on February 28, 2020.^[12]^ Since then, the number of cases has increased continuously. In Mexico, an emerging economy, the government's strategies to diagnose and treat COVID-19 were contained in the document “Algoritmos internos para la atención del COVID-19.”^[13]^ In this document, the diagnostic approach is mostly clinical, indicating that the polymerase chain reaction (PCR) COVID-19 test is mandatory only in those cases with severe symptoms of COVID-19. Of the ambulatory cases, only 10% will be tested, with the rest sent home on isolation restrictions and symptom severity monitoring. All public hospitals adopted this algorithm; however, in the private setting there is adequate test availability to test patients for COVID-19 who have symptoms described in the algorithm dictated by the federal government.^[13]^ In order to direct and protect orthopaedic surgeons and their patients, FEMECOT (Federación Méxicana de Colegios de Ortopedia y Traumatología), the national orthopedic association in Mexico, published their recommendations on trauma care service delivery during the pandemic.^[14]^

### 2.1 Organizational actions made to trauma services

FEMECOT's recommendations included those on outpatient and emergent patient care, operating room guidelines, and academic activities, and are summarized as follows.^[14]^

### 2.2 Outpatient care

To minimize patient-physician contact and reduce patients’ exposure, surgical follow-ups and external consultations should be done with the help of telemedicine as much as possible.^[15,16]^ All nonurgent face-to-face consultations should be postponed. When face-to-face consultation is necessary, the following recommendations should be followed:

1. Patients should come into the examination room alone; in cases where the patient needs help or is a minor, only one relative should join them. Face masks should be universally worn by everyone.
2. Times between appointments should be increased to avoid waiting room crowding.
3. Physical examinations should be focused on the affected area.
4. All patients should be asked for respiratory symptoms and temperature checks should be performed in all the persons coming in for an appointment.
5. The use of methylprednisolone should be avoided, due to its association with increased viral release from hosts.^[17]^

#### 2.2.1 Emergency care

**Figure 1:**
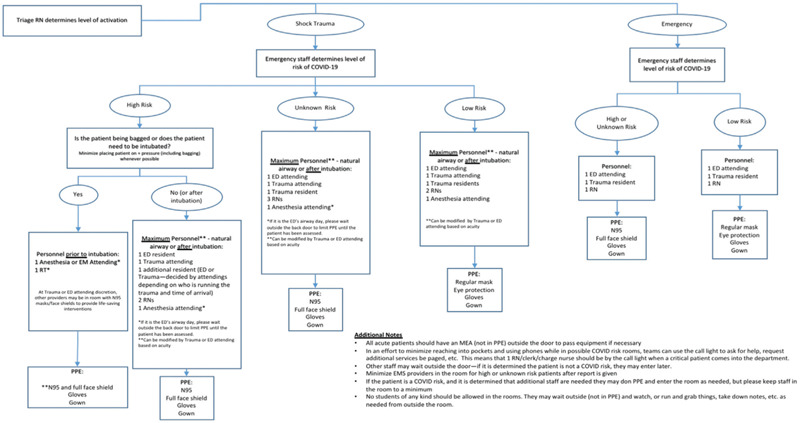
Personal protection equipment in emergency care during COVID-19 pandemic. (Chart modified from the original from Zuckerberg San Francisco General and Trauma Hospital).

Emergency cases must be treated timely, and appropriate PPE must be used to prevent infection, and all patients should be considered as potentially having COVID-19 (Fig. 1). Splints should be favored over casts to minimize the patient return visits to the hospital. In high-risk patients, conservative treatment of certain conditions should be considered if functional outcome is not severely affected and when reconstruction surgery is a possibility when the infection rates permit.^[5,6]^

#### 2.2.2 Operating room

**Figure 2:**
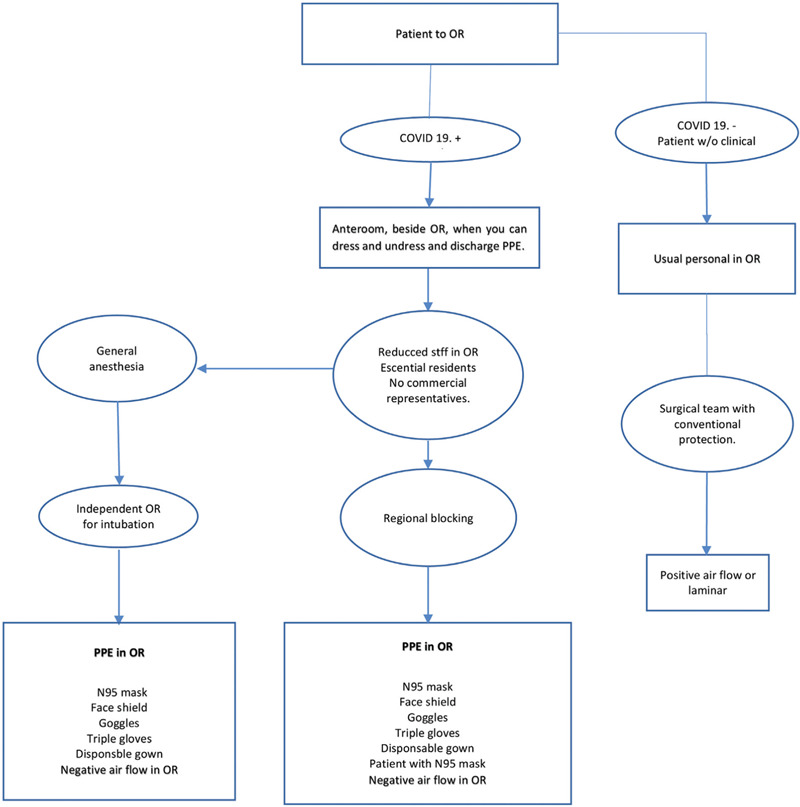
Personal protection equipment in operating room during COVID-19 pandemic. (chart modified from the original from Zuckerberg San Francisco General and Trauma Hospital).

All elective surgeries in patients with high-risk factors that include age greater than 60 years, diabetes, high blood pressure, pulmonary diseases, cancer, cardiovascular disease or immunocompromised patients should be postponed.^[9,10]^ The use of appropriate PPE in the operating room (Fig. 2) is essential to avoid viral transmission during the surgical procedure. Ideally, all patients in a nonurgent/emergency setting must be tested with a PCR COVID-19 test prior to the procedure. However, if the test is not available, protective measures (Fig. 2) should be taken, with the assumption that all patients are COVID-19 positive.^[18–20]^

Operating rooms should be divided into those for COVID-19-positive or suspicious patients and the others for negative patients. In addition to the evidence that transmission can occur not only through aerosol sprays, direct contact, and contaminated fomites, but also with the use of electrocautery, high power tools, and with room air conditioning without negative pressure, the use of this equipment should be minimized.^[11,20]^ General anesthesia should be avoided, instead using regional anesthetic blocks, when possible. During surgery, the patient must use a N95 mask. When general anesthesia is performed, it should happen in a separate room, with the minimum number of necessary personnel.^[21]^ The skin closure should be done with absorbable sutures to avoid the need for suture removal, with telemedicine used for postoperative visits when possible.^[16]^

#### 2.2.3 Academic activities

All academic activities and meetings should be done through teleconferences and online webinars whenever possible.

### 2.3 Effects on number of trauma cases

Stay-at-home orders decreased the number of trauma cases seen at most hospitals, except for hip fractures, which remained constant. Hospitals have also redistributed their resources to focus on managing COVID-19 patients and reserving beds and personnel for COVID-19 care. FEMECOT developed a chart to assist in the prioritizing cases based on their classification of urgency and severity (Table 1). Hospital stays and patient exposures should be minimized, and patients have been hospitalized for as short of a stay as possible. In the public setting, where there are fewer resources, one strategy to achieve these goals is to schedule cases, including semiurgent cases, to have the patients come the same day of the surgery.

**Table 1:** Categorization of case urgency

Priority	Case type
Emergency < than 1 h	Injuries that put the life at risk, acute vascular injury, compartment syndrome, necrotizing fasciitis, pelvic bleeding fracture.
Urgent < than 24 h	Septic arthritis, open fracture, hip or femoral fracture, acute nerve or spinal cord injury, deep wound infection.
Elective urgent < 2 wk	Wound closure, closed fractures, spine fractures without neurological damage, acetabular fractures.
Essential 1–3 mo	Reconstruction surgery, if a surgery needs to be done in a COVID-19-positive patient.
Discretionary more than 3 mo	Arthroplasty surgery, sport injuries^[10]^

In cases where the preop COVID-19 test comes out positive for COVID-19, the patient is evaluated for conservative management or delay of the case as these patients have a high reported mortality rate of 20.5% and intensive care unit (ICU) admission rate of 44.1%. In positive or highly suspicious patients, urgent surgery should be done with the minimum number of personnel in the operating room and proper PPE (Fig. 2).

As health care professionals during this health emergency, we have to remember that services cannot be denied to any patient. The penalty in Mexico is described in articles 470, 471, and 472 of the General Health Law.^[22]^ Additionally, based on Article 132 of the Federal Labor Law, it is specified that employers are obliged to provide workers with the necessary instruments for their work.^[23]^ Further, health care facilities must maintain a complete clinical record that adheres to the Mexican official standards, including informed consent for patient care and whether telemedicine was used.^[24]^

## 3 Other important/unique experiences and lessons learned

The pandemic has changed the way clinical practice and medicine is performed. The literature regarding best practices for management continues to evolve, as will the national government and professional society recommendations. In all the cases, the goal of care is to preserve life over function or the extremity and to continue to be highly vigilant during the pandemic to protect providers and patients through proper safety guidelines.

## 4 Argentina

On March 3, 2020, the first case of COVID-19 was confirmed in Argentina. According to 15465 law, the identification of suspected cases of COVID-19 requires the immediate mandatory notification to the National Health Surveillance System. On March 12, the Argentine President signed a Decree of Necessity and Urgency (DNU). DNU 297/2020 establishes rules and measures to stop the spread of the virus. At the time of writing this report, by June 17, there were 35,552 confirmed cases and 913 deaths (2.5% mortality rate) in Argentina. These figures include 13 cases from the Falkland Islands that were included according to journalism information. The mortality rate was higher in people over the age of 60 years (78%).

At the moment, community spread has been detected in Buenos Aires City, Gran Buenos Aires and Chaco province, in addition to local transmission in Santa Fe, Córdoba, and Rio Negro. In the cities of Buenos Aires and Gran Buenos Aires, where approximately 13 million people live, currently there remains a surge stage of the disease, third stage of the pandemic, with failure to advance to the next stage.

### 4.1 Organizational actions made to trauma services

Because the pandemic first impacted Asian and European countries, Argentina had the opportunity to make political decisions, mandates, and regulations to mitigate the impact of the pandemic based on previous experiences, including approaches to “flatten the curve,” prepare for patient care in the critical surge periods, and avoid collapse of the national health system.

Argentina currently has 4.5 hospital beds and 3.9 doctors for every 1000 inhabitants, although these are irregularly distributed. There are 25 ICU beds for every 100,000 inhabitants. In the case of intensive care beds the actual proportion is 25 beds for every 100,000 inhabitants. At the time of writing this manuscript, the health system has approximately 60% bed occupancy.

Given the expected increase in consultations, hospitalizations, and deaths and considering the experience of other countries, the National Ministry of Health took actions in order to: ^[25,26]^

Strengthen the capacity of health care systems to ensure the rapid detection and diagnosis of COVID-19 cases

- Implement measures to protect staff, patients, and other contacts from exposure
- Develop an Infection Control Committee
- Limit the number of visits for admitted patients in health centers
- Reschedule health care procedures as possible
- Allow fluid communication of the outcomes of these directives
- Evaluate system responses, review established plans and procedures
- Monitor the availability of critical care beds
- Secure routes and forms of transportation for critically ill patients
- Enable availability of public telephone line 148 throughout the territory for 24-hour responses to COVID-19-related questions
- Create a hospital network for patients with “acute respiratory processes” with “febrile units”
- Implement the triage of patients in patient admission areas to identify and attend to suspicious cases.^[27]^

#### 4.1.1 Surgical treatments at the traumatology services during the pandemic

Since March 12, when DNU 297/2020 was signed, most of the health centers in large cities temporarily suspended all scheduled surgeries. However, the term “elective surgery” has had subjective interpretation among surgeons.^[28]^ Orthopedic departments’ leadership has had to set case priorities, taking into account hospital bed demands, PPE availability, and other limited resources.^[29]^

The experience in Singapore with the current COVID-19 crisis has already been documented.^[6]^ Length of stay (LOS) has been used as a metric to determine which procedures should be performed during this crisis.^[30]^ However, deciding which surgeries to postpone has been a topic of controversy. The AAOT (Argentine Association of Orthopedics and Traumatology) has provided general management recommendations regarding this issue, establishing priority levels for different surgeries, mainly based on the which procedures can be postponed without affecting patient outcomes. The AAOT recommendations subdivide orthopaedic surgeries into 5 levels.^[31]^

#### 4.1.2 Elective/programmed surgery (Level 1)

Patients with chronic diseases in whom the surgery can be delayed for more than 3 months without significant health damage. Examples are joint replacements, spinal arthrodesis, chronic rotator cuff tears, and degenerative meniscal tears. These types of surgeries should be postponed until the COVID-19 pandemic is under control.

#### 4.1.3 Delayed short-term surgery (Level 2)

Chronic diseases for which surgery could be delayed for not more than 3 months without affecting the final result. Anterior cruciate ligament reconstructions, acute rotator cuff injuries, or decompressions in myelopathies would be examples of conditions in this category. If possible, these procedures should be performed on an outpatient basis to minimize resource utilization.

#### 4.1.4 Trauma relative urgency (Level 3)

Traumatic musculoskeletal injuries that should be performed prior to 2 weeks to avoid further damage or poor results. Examples of these injuries are tibia fractures, wrist fractures, and any fractures where failure to repair the injury would result in increased morbidity.

#### 4.1.5 Trauma urgency (Level 4)

Traumatic injuries that must be addressed within 48 hours. These include femur fractures in polytrauma patients and hip fractures in elderly patients.

#### 4.1.6 Emergency (Level 5)

Life or limb threatening should be taken for surgery with an objective of minimizing the need for ventilator assistance and avoiding the use of blood banks and ICU beds. Examples of these injures include acute vascular injury, acute compartment syndrome, and open fractures.

Depending on the local COVID-19 infection rates, each regional Orthopedic Department or program may change which levels of surgery are appropriate. The AATO's position is that each service is positioned to select amongst the categories based on the needs and priorities of their region and hospital system.

#### 4.1.7 General recommendations in the surgical field

To reduce exposure to COVID-19 during surgery, the current recommendations from the AATO are

- Reduce the use of procedures or techniques that can produce infectious aerosols when possible.
- Minimize the number of people in the operating room, especially when endotracheal intubation is being performed.
- Use levels of surgery criteria to make decisions about the cancellation or postponement of the surgical procedure.
- Follow all recommended preventive measures to reduce the possibility of viral transmission amongst healthcare workers.
- Utilize full PPE (N95 mask, Cap, eye protection, and surgical gown blood repellent) for those cases in which testing cannot be performed or the results will return after the procedure will need to be started.
- Use standard PPE (regular surgical mask, cap, eye protection, and blood repellent surgical gowns) for those patients with negative tests.^[32–34]^

#### 4.1.8 Preoperative screening

At the time of writing of this section, there were no generally established guidelines for preoperative screening for COVID-19. Guo et al^[34]^ recommended that patients undergo testing for COVID-19 before surgery if possible, with mask placement on patients at all times. Another study from China evaluated 34 elective orthopaedic surgery patients who were in the incubation period of COVID-19. These patients had surgery without having been identified preoperatively as having COVID-19. A large proportion of these patients had major complications with 20.5% mortality rates and a 44.1% rate of patients requiring an ICU bed.^[35]^ The main reasons to identify patients with COVID-19 prior to orthopaedic surgery are to avoid these complications and appropriately protect healthcare staff.^[36]^ Recommended presurgical protocols are as follows:

- Telephone contact with COVID-19 screening questions and verification of presurgical studies. The patient is asked to perform a simple smell test with vinegar and coffee.
- Additional presurgical studies that include testing for COVID-19 by PCR, rapid immunochromatography tests for IgM and IgG, and updated chest X-ray.
- Secondary triage questions for surgical patients being admitted that include questions regarding residence, symptoms, close contacts, and work situations.^[27]^
- Prior to surgery, patients should be evaluated by an anesthesiologist, who will repeat the screening questions. Patients are held in locations based on their COVID-19 testing status.

#### 4.1.9 Internal organization of trauma services

The decisions made by different trauma services in our country differ markedly. This is because the economic, material, and human resources vary between important cities such as Buenos Aires, Córdoba, Rosario, and others areas of the country. Additionally, the number of infections in the country varies enormously according to the different provinces (more than 90% of the infections occurred in the Buenos Aires and Gran Buenos Aires areas). The main health centers in Buenos Aires chose to divide the trauma service personnel into 2 balanced work groups that rotated every 14 days. The reason for this was 2-fold; it reduced the potential exposure of staff as well as the risk of cross-contamination across the staff. This redistribution of personnel left an adequate number of staff to cover the trauma demand, particularly given that elective surgeries were suspended and that there were fewer trauma patients resulting from traffic accidents.

Social distancing of medical staff includes cancelling classes, telecommuting with flexible worksites, replacing in-person conferences with teleconferences, screening for COVID-19, restricting hospital visitors, and limiting interpersonal contact of healthcare workers with proper protection and 2-m distancing when possible.^[16]^ In some places, health personnel who do not require direct patient contact worked from home, some staffing the general call line for mandatory reporting of suspected cases (telephone line 148). In areas with few COVID-19 cases, most health centers continued to work as they did pre-pandemic, since the amount of trauma had not changed from pre-pandemic levels.

## 5 Other important/unique experiences and lessons learned

The Argentinian experience was consistent with previous reports illustrating the importance of preventing health personnel infections by respecting all regulations and applying the correct use of PPE.^[10]^ The consequences of COVID-19 infections in the healthcare work force are problematic, not only because they can infect vulnerable people, but also they are required for patient care during the pandemic.^[8]^ In addition to the morbidity, the pandemic will leave behind a devastated economy at all levels, with significant consequences for the general public health of Argentina. The expectation is that the entire scientific and healthcare communities can join to address what is yet to come.

## 6 Colombia

The prevention of COVID-19 has affected the economy, social balance, and scientific community, like it has in the rest of the world. In an effort to contribute to the overall health of society, Colombian Society of Orthopaedic Surgery and Traumatology (Sociedad Colombiana de Cirugía Ortopédica y Traumatología, SCCOT) and Colombian Orthopaedic Trauma Society (Sociedad Colombiana de Trauma, SOCOT) have provided information, training, analysis, interpretation, and education on what has been learned about COVID-19 and decision making in the management of patients with musculoskeletal conditions.

Colombia has a population of 50 million habitants.^[37]^ The first reported COVID-19 case was on March 6.^[38]^ Since then, there have been more than 70,000 cases, 2,000 deaths, and 38,000 recovered patients through June.

### 6.1.1 Organizational actions made to trauma services

The Colombian Orthopedic Societies have performed a search of current evidence regarding the organization of orthopaedic surgeries and trauma services, the management of patients, and the protective measures for the health personnel during the COVID-19 pandemic. The search was carried out in the PUBMED and Scielo databases and the Colombian government health institutions and scientific societies. Recommendations were made for the different types of orthopaedic practices: outpatient clinics, inpatient hospitalizations, surgery (elective and emergency), and specific subspecialty recommendations for spinal surgery, trauma, hand surgery, and pediatric orthopaedics.^[39]^

Scheduled elective surgeries were suspended or delayed as they were not vital, with only urgent surgeries being performed. PPE use was recommended for the health care staff and patients at all times. Patients with multiple trauma and complex injuries require continued comprehensive management and stabilization in the emergency, trauma, and subspecialty departments. All patients are required to undergo PCR COVID-19 testing and preanesthetic evaluation, which includes a telephone questionnaire and preventative recommendations prior to surgery. When intubation and aerosol-generating procedures are performed, all appropriate PPE must be used.^[40]^ Although outpatient consultations are often required to evaluate patients, telemedicine consultations have been increasingly employed for many visits. Further, only the necessary personnel required to treat patients should be directly involved with their care, including trainees. Smaller subsets of the surgical teams were created and rotated to protect against cross-contamination of the teams.

At the educational level, SCCOT implemented a Virtual Convention Center (https://vcccolombia.com/) to have a state-of-the-art technology platform and to continue sharing knowledge with the 10 SCCOT-affiliated societies. Webinars also have been held with the participation of international colleagues, including those from the United States of America, (USA), Spain, and Colombia sharing our experiences. The planning and organization of academic and administrative SCCOT events and CLEMI (Center for Latin American Research and Minimal Invasive Surgery) have been impacted by COVID-19, due to social distancing requirements, with multiple postponements and cancellations.

The technology used to develop SCCOT's Virtual Convention Center will also be directed toward creating telehealth options for its membership. These include a variety of innovative telehealth solutions, including tele-orientation, tele-support, and telemedicine in many different forms (interactive, noninteractive, tele-expertise, and tele-monitoring). The expectation is that these solutions will improve patient care options through different digital platforms.

## 7 Other important/unique experiences and lessons learned

The clinical practice created in response to the pandemic has significantly affected every aspect of the orthopaedic surgery practice. Successful adaptations have been created, allowing for improvements in patient care that are likely to last beyond the end of the pandemic. These novel initiatives have been consistently directed toward improving the health care approach in Colombia, and are consistent with SCCOT's and SOCOT's academic and scientific missions.^[40]^

## 8 Brazil

Brazil is a large country with a population of over 212 million inhabitants. The first patient with a confirmed diagnosis of a COVID-19 infection occurred on February 28, 2020. From February 28, until through July 7, Brazil had reached a total of more than 1,674,000 infected patients with almost 66,900 deaths, and 1,072,000 recovered patients (Table 2). COVID-19 infections evolved sequentially in Brazil, with several states in different stages of contamination and having different numbers of cases throughout the pandemic. There are also significant differences in the public health care structure between states and municipalities, some with more optimal structures that have adequate numbers of intensive care beds, human resources, and therapeutics, while others have severe structural deficiencies.

**Table 2:** Numbers of confirmed, deaths, and recovered patients

	Confirmed	Deaths	Recovered
Brazil	1,674,655	66,868	1,072,229
State of Sao Paulo	332,708	16,475	—
City of Sao Paulo	142,502	7,743	—

Source: www.seade.gov.br.

### 8.1 Organizational actions made to trauma services

Sao Paulo, the largest city in Brazil, developed measures that were representative of those responses. These actions included:

- Restrictions on the movement of vehicles and people
- Recommendations for social distancing and stay-at-home orders
- Closure of all nonessential activities
- Use of masks
- Construction of 3 field hospitals with about 2100 hospitalization beds for patients with moderate and severe COVID-19 symptoms
- Increase of intensive care beds in the public health system
- Adequacy of the Hospital das Clinicas, Faculty of Medicine, University of Sao Paulo (HCFMUSP) to be the reference of the public hospitals for the treatment of patients severely affected by COVID-19.

### 8.2 Preparation of the Hospital das Clínicas Complex

The Hospital das Clinicas, Faculty of Medicine, University of Sao Paulo (HCFMUSP), is a hospital complex located in the city of São Paulo with about 2400 beds distributed across 6 specialty institutes and 2 long-stay hospitals. The HCFMUSP Complex is part of the hierarchical municipal and state emergency care system and is responsible for the management of high complexity cases in the Sao Paulo city region with a population of about 4.5 million inhabitants.

The Central Institute (ICHC – the complex's main building) was reserved exclusively for this purpose, allocating 500 inpatient beds for COVID-19 patients with moderate-to-severe symptoms, and 300 intensive care beds for critically ill patients, including those who required mechanical ventilation.

The following measures were necessary to achieve this target:

- Relocation of hospitalized COVID-19 patients to the ICHC, and multidisciplinary teams responsible for treating these patients from other institutes of the Hospital das Clinicas Complex
- Suspension of elective surgeries, with the performance of only oncological procedures, urgent and emergency surgeries, and organ transplants; these surgeries were performed in the other institutes of the Complex
- Drastic reduction in the number of outpatient consultations in all units of the Complex to reduce the circulation of people and minimize the possibility of cross-contamination
- All patients who tested positive or who were considered highly suspected of having COVID-19 were admitted to the ICHC
- All human resources, diagnostics, therapeutics, multidisciplinary teams, equipment, PPE, and other equipment essential for the treatment of COVID-19 patients were available in the ICHC.

### 8.3 Organization of institute for orthopaedics and traumatology (IOT)

The IOT, which is dedicated to the treatment of patients with musculoskeletal conditions, had 97, or approximately 60% of its inpatient beds occupied by patients from other specialties that included internal medicine, geriatrics, rheumatology, neurology, infectious diseases, and palliative care. This left the remaining beds for patients with musculoskeletal conditions. The IOT policies included the following:

- All patients transferred to or seen for consultation at the IOT were screened and tested negative for COVID-19.
- No visits to hospitalized patients were permitted.
- In situations in which the presence of a companion was necessary (in accordance with Brazilian legislation), the individuals were required to wear protective masks, with exchanges of the companion only allowed every 24 hours.
- Mandatory use of masks by all health care professionals and administrative employees in inpatient, outpatient, and emergency wards.
- Establishment of an orthopaedic trauma team (5 attendings and 2 residents) exclusively dedicated to the treatment of COVID-19 patients hospitalized at ICHC.
- The IOT emergency unit addressed emergency orthopaedic consultations, including those referred with medium and high complexity traumatic conditions referred according to the Municipal and State Regulation of Urgency and Emergency.
- Those patients who present respiratory symptoms after admission are isolated in a specific area of the IOT reserved for suspected patients, tested by PCR, and undergo preventive isolation methods.

The policies for orthopaedic surgeries performed at the IOT on COVID-19-negative patients included:

- Mandatory use of N95 mask and face-shield by all team members
- Protection of all essential equipment for the operating room, with nonessential equipment not brought into the active care areas
- A nursing technician located both inside and outside the operating room to provide the necessary support for the material and medication requests
- Terminal cleaning of the operating room after surgical procedures

### 8.4 Impact on the patient care indicators at the Institute of Orthopedics and Traumatology

#### 8.4.1 Emergency unit visits

Compared with a comparable period in 2019, there was approximately a 52% decrease in the total number of visits from March to May 2020. During the months of April and May, 2020, there was a 61% average reduction in the total number of visits.

#### 8.4.2 Number of urgent and emergency hospitalizations

In March 2020, there was an approximately 5% increase in emergency hospitalizations due to the number of cases that occurred in the first half of that month. Due to the decrease in traffic accidents and the movement of people, in April 2020, there was a drop of about 41% in emergency hospitalizations. In May, the decrease in hospitalizations was 25%, lower than the previous month, correlating with rates of isolation. From March to May 2020, the number of hospitalizations was about 21% lower compared with the same period in 2019.

#### 8.4.3 General orthopaedic surgeries

Approximately 500 orthopaedic surgeries had been performed monthly at the IOT prior to the beginning of the pandemic. With the suspension of elective surgeries and maintenance of only oncological, urgent, and emergency surgeries, there was a 48.9% reduction in the total number of surgical procedures performed at the institute. In May 2020, the number of surgeries was 66.5% lower compared to the same period in 2019.

#### 8.4.4 Orthopaedic trauma urgent and emergency surgeries

Orthopaedic trauma urgent and emergency surgeries decreased by 28.5% compared with the same months in 2019, excluding those surgeries related to spinal trauma and hand and microvascular surgery. In March 2020, the impact was small, with a 4.3% reduction. However, in May, the decrease was about 45% compared with the same month in 2019.

## 9 Other important/unique experiences and lessons learned

The experience in Sao Paulo reflected that of much of that of the rest of Brazil. Similarly, that experience of the HCFMUSP Complex typified that of other centers in large cities in Brazil. From March 15 through July 7, 2020, 3485 patients diagnosed with COVID-19 were admitted to the ICHC and, during the same period, 2248 were discharged from the hospital. The median length of stay of patients with COVID-19 was 12 days. The infection rate of HCFMUSP-Complex employees by COVID-19 was about 11%. Compared with the same period in 2019, during the months of March through May, 2020, at the IOT there was a significant decrease in the number of emergency room visits, 50% reduction in the total number of surgeries due to the suspension of the majority of elective procedures, and a decrease in the number of surgeries related to orthopaedic trauma.

## 10 Conclusions

During the COVID-19 pandemic, orthopaedic trauma care was still an essential service required for patients in Latin America. Despite not having a universal approach across countries, there were 4 common practices in countries across the region: Proper protection to decrease the risk of transmission between individuals, including patients and healthcare providers; Avoidance of dissemination through isolation and social distancing; Restriction of unnecessary activities, including elective outpatient visits and surgical care, patient flow and visitors to hospitals, and conferencing; and Preservation of resources, including PPE and staffing. The current literature sets guidelines for our continued actions in the face of the COVID-19 pandemic, although future best practices must be verified through improved levels of evidence. Further, while we safeguard life over function, we should continue to evaluate the economic consequences of these adaptive policies, work to effectively manage care during the pandemic, and learn from the experiences of other countries.
